# Spanish registry of hemoglobinopathies and rare anemias (REHem-AR): demographics, complications, and management of patients with β-thalassemia

**DOI:** 10.1007/s00277-024-05694-z

**Published:** 2024-03-23

**Authors:** Eduardo J. Bardón-Cancho, José Manuel Marco-Sánchez, David Benéitez-Pastor, Salvador Payán-Pernía, Anna Ruiz Llobet, Rubén Berrueco, Marina García-Morin, Cristina Beléndez, Leonor Senent, María José Ortega Acosta, Irene Peláez Pleguezuelos, Pablo Velasco, Anna Collado, Marta Moreno-Carbonell, Bienvenida Argilés, Inmaculada Pérez de Soto, María del Mar Bermúdez, Eduardo J. Salido Fiérrez, Adoración Blanco-Álvarez, Pablo González Navarro, Elena Cela

**Affiliations:** 1 Data Manager de Grupo de trabajo de Eritropatología de la Sociedad Española de Hematología y Oncología Pediátricas (SEHOP), Hospital General Universitario Gregorio Marañón, Calle O’Donnell, 48, Madrid, España; 2 Sección de Hematología y Oncología Pediátricas. Servicio de Pediatría. Hospital General Universitario Gregorio Marañón. Instituto de Investigación Sanitaria Gregorio Marañón. CSUR Eritropatología. ERN-EuroBloodNet. CIBERER, Facultad de Medicina, Universidad Complutense de Madrid, Madrid, España; 3 Grupo de Investigación Translacional en Anemias Minoritarias, Unidad de Eritropatología. Servicio de Hematología Clínica. Hospital Universitario Vall d’Hebron, Vall d’Hebron Institut de Recerca (VHIR) y Vall d’Hebron Institut d’Oncologia (VHIO), ERN-EuroBloodNet. CIBERER, Universitat Autònoma de Barcelona. Grupo de Eritropatología SEHH, Grupo Clínico Vinculado GCV21/ER/1, Barcelona, España; 4 Servicio de Hematología. Hospital Universitario Virgen del Rocío, Instituto de Biomedicina de Sevilla (IBIS)-Consejo Superior de Investigaciones Científicas (CSIC), Universidad de Sevilla, Sevilla, España; 5 Servicio de Hematología Pediátrica. Hospital Sant Joan de Déu Barcelona, Institut de Recerca Pediàtrica Hospital San Joan de Déu de Barcelona (IRP-HSJD), Universitat de Barcelona, Esplugues de Llobregat, Barcelona, España; 6 Servicio de Hematología, Hospital Universitario y Politécnico La Fe, Valencia, España; 7 Servicio de Hematología Infantil, Hospital Universitario Virgen de las Nieves, Granada, España; 8 Servicio de Hematología Infantil, Hospital Universitario Vall d’Hebron, Barcelona, España; 9 Servicio de Hematología. Hospital General Universitario Gregorio Marañón. Instituto de Investigación Sanitaria Gregorio Marañón. CSUR Eritropatología. ERN-EuroBloodNet, Universidad Complutense de Madrid, Madrid, España; 10 Servicio de Hematología Infantil, Hospital Universitario y Politécnico La Fe, Valencia, España; 11 Servicio de Oncología Pediátrica, Hospital Virgen de la Arrixaca, Murcia, España; 12 Servicio de Hematología, Hospital Virgen de la Arrixaca, Murcia, España; 13 Unitat de Genètica Molecular Hematològica. Servei d’Hematologia, Hospital Universitari Vall d’Hebron, Murcia, España; 14 Bioestadístico. Unidad de Investigación Materno Infantil. Fundación Familia Alonso (UDIMIFFA). Instituto de Investigación Sanitaria Gregorio Marañón (IiSGM), Hospital General Universitario Gregorio, Madrid, España

**Keywords:** Registry, Thalassemia, Hemoglobinopathies, Anemia, Complications, Spain

## Abstract

**Introduction:**

The increase in the number of patients with hemoglobinopathies in Europe in recent decades highlights the need for more detailed epidemiological information in Spain. To fulfil this need, the Spanish Society of Pediatric Hematology and Oncology (SEHOP) sponsored the creation of a national registry of hemoglobinopathies known as REHem-AR (Spanish Registry of Hemoglobinopathies and Rare Anemias). Data from the transfusion-dependent (TDT) and non–transfusion-dependent (NTDT) β-thalassemia cohorts are described and analyzed.

**Methods:**

We performed an observational, multicenter, and ambispective study, which included patients of any age with TDT and NTDT, registered up to December 31, 2021.

**Results:**

Among the 1741 patients included, 168 cases of thalassemia were identified (103 TDT and 65 NTDT-patients). Survival at 18 years was 93% for TDT and 100% for NTDT. Regarding management, 80 patients with TDT (77.7%) and 23 patients with NTDT (35.4%) started chelation treatment during follow-up, with deferasirox being the most widely used. A total of 76 patients within the TDT cohort presented at least 1 complication (73.8%), the most frequent being hemosiderosis and osteopenia-osteoporosis. Comparison of both cohorts revealed significant differences in the diagnosis of hepatic hemosiderosis (*p* = 0.00024), although these were not observed in the case of cardiac iron overload (*p* = 0.27).

**Discussion:**

Our registry enabled us to describe the management of β thalassemia in Spain and to analyze the morbidity and mortality of the cohorts of patients with TDT and NTDT. Complications related to iron overload in TDT and NTDT account for most of the morbidity and mortality of the disease, which is associated with a considerable social, psychological, and economic impact, although cardiac, osteopathy and endocrinological complications requiring more attention. The convenience and simplicity of online registries make it possible to homogenize variables and periodically update data, thus providing valuable information on these diseases.

## Introduction

Hemoglobinopathies are genetic disorders of hemoglobin (Hb) and include structural hemoglobinopathies and thalassemia syndromes. Globally, 400,000 newborns are affected annually by clinically significant hemoglobinopathy. Of these, approximately 42,000 have β-thalassemia major (TM) and 14,000 have α-TM [1–5]. Survival of affected patients has improved during recent decades. Neonatal screening and improvements in transfusion support and monitoring and treatment of iron overload, its main complication, have changed the prognosis of the disease throughout the world [1, 4]. The commercial availability of oral iron chelators has improved quality of life. In addition, splenectomy has been relegated to a secondary level, and this has reduced associated complications, such as bleeding, thrombosis, and infections. Hematopoietic stem cell transplantation (HSCT) from a compatible donor is currently the only curative option, pending advances in gene therapy, although it is not widely available [1].

In the last 20 years, largely due to increased migratory movements from endemic areas (Mediterranean countries, sub-Saharan Africa, India, and Southeast Asia), the number of patients with hemoglobinopathies in Europe has increased, to the extent that the condition is now a public health interest [1]. Analyzing the current state of these diseases through registries provides valuable information on prevention and diagnostic strategies, treatment protocols, and health policies, although national registries have only been created in a few European countries (Greece [1, 6], United Kingdom [2, 7], Italy [8], France [9], and Turkey [10]).

In order to collect epidemiological information on patients with hemoglobinopathies in Spain and ensure institutional support, in 2014, the Spanish Society of Pediatric Hematology and Oncology (Sociedad Española de Hematología y Oncología Pediátricas [SEHOP]) sponsored the creation of a national registry, the Spanish Registry of Hemoglobinopathies and Rare Anemias (Registro Español de Hemoglobinopatías y Anemias Raras [REHem-AR]). The first data, which were from pediatric patients only, were published in 2016 [11]. An update was published after the inclusion of adult patients from 2018 in the registry by the Red Cell Disorder Working Group of the Spanish Society of Hematology and Hemotherapy (Sociedad Española de Hematología y Hemoterapia [SEHH]) [12]. REHem-AR currently collects data from patients with rare anemias throughout Spain, including sickle cell disease (SCD), TM, thalassemia intermedia (TI), pyruvate kinase deficiency, glucose 6-phosphodehydrogenase deficiency (G6PD), congenital dyserythropoietic anemias, Blackfan-Diamond anemia, congenital sideroblastic anemias, hereditary xerocytosis, and other clinically significant anemias. In this paper, we report and analyze data from the TM and TI cohorts (i.e., transfusion-dependent thalassemia [TDT] and non–transfusion-dependent thalassemia [NTDT]).

## Methods

We performed an observational, multicenter, and ambispective study. The registry began in January 2014, and was based on only retrospective clinical data collected until that time. Data were then collected prospectively, and follow-up was on an annual basis. We present the results corresponding to patients registered up to December 31, 2021.

All pediatricians and hematologists monitoring patients with rare anemias were invited to participate in the registry through the Red Cell Disorder Groups of the SEHOP and the SEHH. Patients of any age who had had at least 1 consultation at any of the centers were included. Patients were included in the cohort at birth if neonatal screening was available or on the date of diagnosis if it was performed subsequently for another reason, when medical records started to become available. Universal neonatal SCD screening began in 2003 in Madrid and progressively in the rest of Spain until the whole country was covered in 2021. Although TM is not an objective of the program, it is detected through the absence of HbA.

Informed consent was obtained from all patients or their legal guardians, in accordance with the Declaration of Helsinki.

The recording and analysis of the data were approved by the local ethics committees, by the Prosecutor’s Office for Minors, and by the Spanish Data Protection Agency (Agencia Española de Protección de Datos [AEPD]) and reported to the Spanish Agency of Medicines and Medical Devices (Agencia Española del Medicamento y Productos Sanitarios [AEMPS]).

The variables were entered in a pseudonymized manner into the REDCap web application (Version 6.12.0, © 2023 Vanderbilt University) by the treating physicians or by a common data manager and included personal data, date of birth, sex, diagnosis and date thereof, reason for diagnosis, country of birth, genotype, results of imaging tests, clinical complications, treatments, and follow-up data (alive or deceased, loss to follow-up and reasons).

Duplicate registration of the same patient (generally due to contact with more than 1 center) was detected through the patient identification code of the Spanish National Health System when available; in certain cases, the combination of the patient’s name, sex, date of birth, and personal contact with the treating physician were necessary to identify the duplication.

The registry is based on a network that helps to maintain personal contact between all physicians treating patients with hemoglobinopathies to ensure high-quality care and sharing of experiences. This collaboration has generated the publication of national clinical practice guidelines for the treatment of both TM and TI [13] and SCD [14], thus reinforcing the acceptability of this registry.

### Definition of variables

Patients with TDT were defined as those who requiring lifelong regular blood transfusion to survive (or at least 8 units per year in patients diagnosed before the generalization of the new terms), and without adequate transfusion support, they would experience complications and have a short life span. An example would be patients with β-TM or severe Hb E/β-thalassaemia, as defined by the current guidelines [15–17]. In contrast, patients with NTDT do not require lifelong regular transfusions for survival, although may require occasional or intermittent transfusions, such as patients with β-TI or mild-moderate Hb E/β-thalassaemia [18, 19]. The main complications of both groups were defined according to current management guidelines and they are well documented [16–19]: in the case of iron overload, it is based in relation to liver iron levels by T2* MRI or by ferritin levels if MRI had not been available; cardiovascular disease as the presence of hypertension, arrhythmias or signs of heart failure; osteopenia has been defined as a Z-Score ≤ − 2 SD in bone densitometry; the presence of a height < 2 SD according to the age and sex of the patient; or the presence of hormonal alterations or alloimmunization phenomena, among others.

### Statistical analysis

The analysis was performed using R Statistical Software (version 4.2.1; R Foundation for Statistical Computing, Vienna, Austria). The descriptive analysis was carried out according to the type of variable. Absolute and relative frequencies were used for qualitative variables. The associations of interest between these variables were studied using Pearson’s chi-square or Fisher’s exact test, as appropriate. Quantitative variables were reported as median and interquartile range. Hypotheses were tested using the Wilcoxon signed-rank test for comparisons between 2 groups, while the Kruskal-Wallis test was used in those cases where the number of groups was greater than 2. The probability of survival in years from birth to death or last follow-up visit was described using Kaplan-Meier curves, stratifying by disease or clinical group. Hypotheses between survival curves were tested using the log-rank test. Statistical significance was set at *p* < 0.05.

## Results

**Fig. 1:**
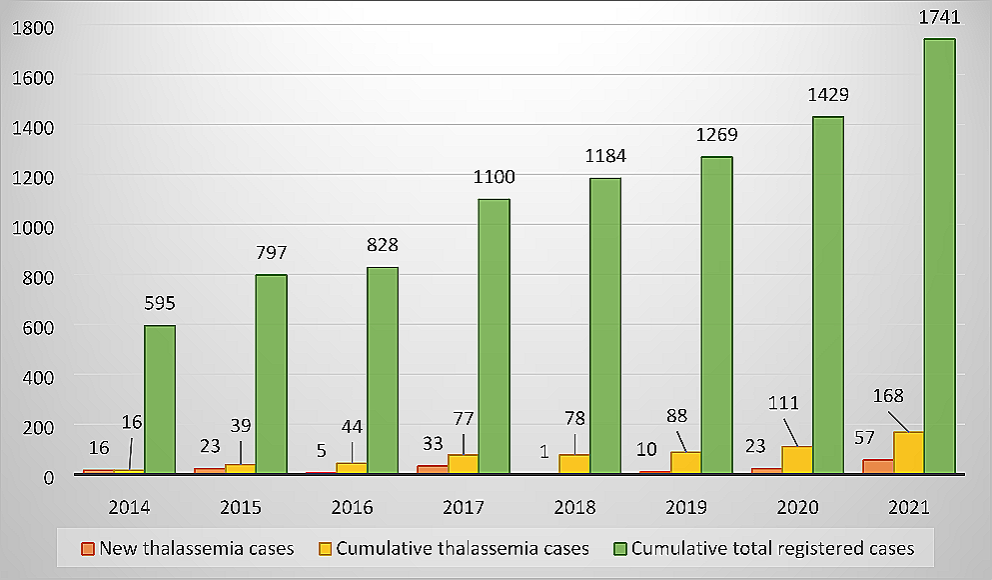
Total registered patients and patients with a diagnosis of thalassemia per year and accumulated

A total of 1741 patients with red cell disorders currently included in REHem-AR were collected from 78 hospitals throughout Spain. The annual and cumulative records are shown in Fig. 1. A total of 181 patients with TDT or NTDT were registered by 24 centers. Thirteen records were excluded owing to duplication of patients, with a total of 168 analyzable records and a median of 2 records per center (1–38). Of these, 103 patients were TDT (61.4%) and 65 NTDT (38.6%). The demographic characteristics are shown in Table 1. The autonomous communities that registered the most cases were Catalonia (35.7%) and Andalusia (25.0%), followed by the Community of Madrid (14.3%) and the Valencian Community (13.1%).

**Table 1:** Demographic characteristics of patients with transfusion-dependent (TDT) and non–transfusion-dependent thalassemia (NTDT)

Phenotype	TDT	NTDT	Total
Number of patients (%)	103 (61.3)	65 (38.7)	271 (100)
Ratio men/women	1.24	0.62	0.92
Alive (%)	66 (64.1)	59 (90.1)	197 (72.7)
Deceased (%)	4 (3.9)	1 (1.5)	6 (2.2)
Lost to follow-up (%)	33 (32.0)	5 (7.7)	68 (25.1)
Age at diagnosis (years)	0.63 (0.25, 1.51) [0.00-6.03]	6.78 (1.50, 27.88) [0.00-64.99]	1.00 (0.20, 3.92) [0.00-64.99]
Current age (years)^1^	13.86 (7.23, 20.06) [0.23–47.70]	31.75 (14.79, 46.78) [0.28–69.58]	12.63 (6.05, 22.46) [0.00-69.58]
Length of follow-up (years)	13.29 (6.68, 18.33) [0.04–46.70]	8.77 (5.05, 19.33) [0.28–69.58]	7.92 (3.82, 15.67) [0.00-69.58]
Main countries of birth of the patient	Spain: 64 (62.1) Morocco: 7 (6.8) Pakistan: 7 (6.8) Italy: 4 (3.9) Romania: 4 (3.9)	Spain: 38 (58.5) Morocco 3 (4.6) Italy: 3 (4.6) India: 3 (4.6)	
Main countries of birth of the father	Spain: 32 (31.1) Morocco: 23 (22.3) Pakistan: 9 (8.7) Italy: 5 (4.8) Romania: 5 (4.8)	Spain: 25 (38.5) Morocco: 8 (12.3) Pakistan: 4 (6.1) Algeria: 2 (3.1) Colombia: 2 (3.1) Romania: 2 (3.1)	
Main countries of birth of the mother	Spain: 33 (32.0) Morocco: 23 (22.3) Pakistan: 9 (8.7) China: 5 (4.8) Romania: 5 (4.8)	Spain: 25 (38.5) Morocco: 8 (12.3) Pakistan: 4 (6.1) Algeria: 2 (3.1) Colombia: 2 (3.1) Romania: 2 (3.1)	

Data for qualitative variables are expressed as absolute frequency and percentage. Quantitative variables are expressed as the median (interquartile range) [data range].

^1^Only for active patients.

### Transfusion-dependent thalassemia

The main reason for diagnosis was clinical anemia (77.7%). SCD was diagnosed in 11.6% of affected patients at birth in neonatal screening. More infrequently, cases were diagnosed via the family study (5.8%) and the adoption study (1.9%), and 3.0% were diagnosed via other, uncategorized sources. β-Globin gene sequencing was performed in 62.1% of patients (Table 2), with 17 mutations recorded, although 90% of all mutated alleles are associated with the 9 most common mutations. This study was not considered necessary in 23 patients (22.3%). Of the 41 patients (39.8%) in whom the α-globin gene study was performed, associated deletion was observed in 4.9% of cases. Testing for G6PH deficiency (30.1%) revealed no cases.

**Table 2:** *HBB* mutations registered in the cohort of patients with transfusion-dependent β-thalassemia (TDT)

Classical nomenclature	HGVS nomenclature	Allelic phenotype	Homozygous state	Heterozygous state	Total mutated alleles	Allele frequency (%)
Codon 39 (C > T)	*HBB*:c.118 > T	β^0^	10	14	34	31.2
IVS-I-110 (G > A)	*HBB*:c.93-21G > A	β^+^	6	10	22	20.2
IVS-I-1 (G > A)	*HBB*:c.92 + 1G > A	β^0^	4	5	13	11.9
Codons 8/9 (+ G)	*HBB*:c.27_28insG	β^0^	3	1	7	6.4
Codon 6 (-A)	*HBB*:c.20delA	β^0^	1	3	5	4.6
Codon 5 (-CT)	*HBB*:c.17_18delCT	β^0^	1	3	5	4.6
IVS-I-5 (G > C)	*HBB*:c.92 + 5G > C	β^+ (severe)^	2	0	4	3.7
IVS-I-6 (T > C) (Portuguese type)	*HBB*:c.92 + 6T > C	β^+^	1	2	4	3.7
IVS-II-745 (C > G)	*HBB*:c.316-106 C > G	β^+^	0	4	4	3.7
IVS-II-849 (A > C)	*HBB*:c.316-2 A > C	β^0^	1	0	2	1.8
Codon 8 (-AA)	*HBB*:c.25_26delAA	β^0^	1	0	2	1.8
IVS-I-2 (T > G)	*HBB*:c.92 + 2T > G	β^0^	0	2	2	1.8
IVS-II-844 (C > G)	*HBB*:c.316-7 C > G	β^+^	0	1	1	0.9
IVS-II-1 (G > A)	*HBB*:c.315 + 1G > A	β^0^	0	1	1	0.9
Hb Monroe	*HBB*:c.92G > C	β^0^	0	1	1	0.9
-28 (A > G)	*HBB*:c.-78 A > C	β^+^	0	1	1	0.9
Codons 41/42 (-TTCT)	*HBB*:c.126_129delCTTT	β^0^	0	1	1	0.9

HGSV: Human Genome Variation Society.

Slightly more than two-thirds of patients (36%) were followed up in more than 1 health center. Losses to follow-up for reasons other than death were due to a change of center in 54.5% and migration to another country in 36.4%.

**Fig. 2:**
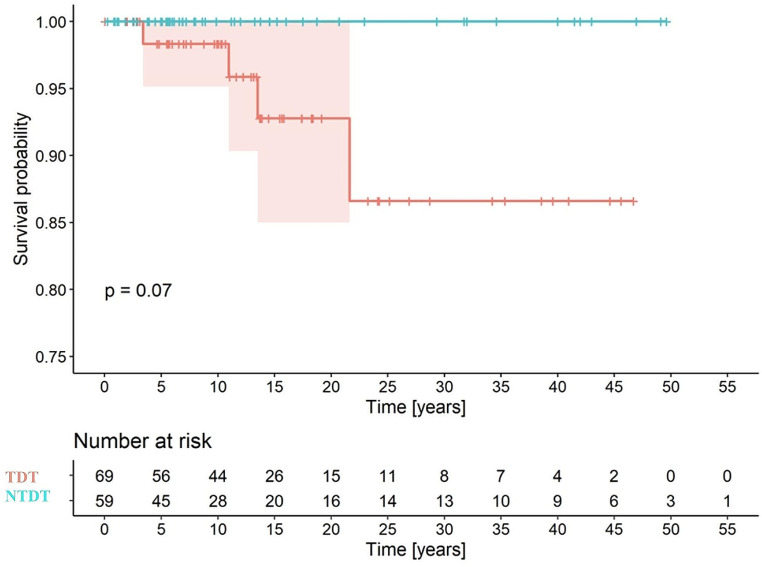
Survival curve based on diagnosis

**Fig. 3:**
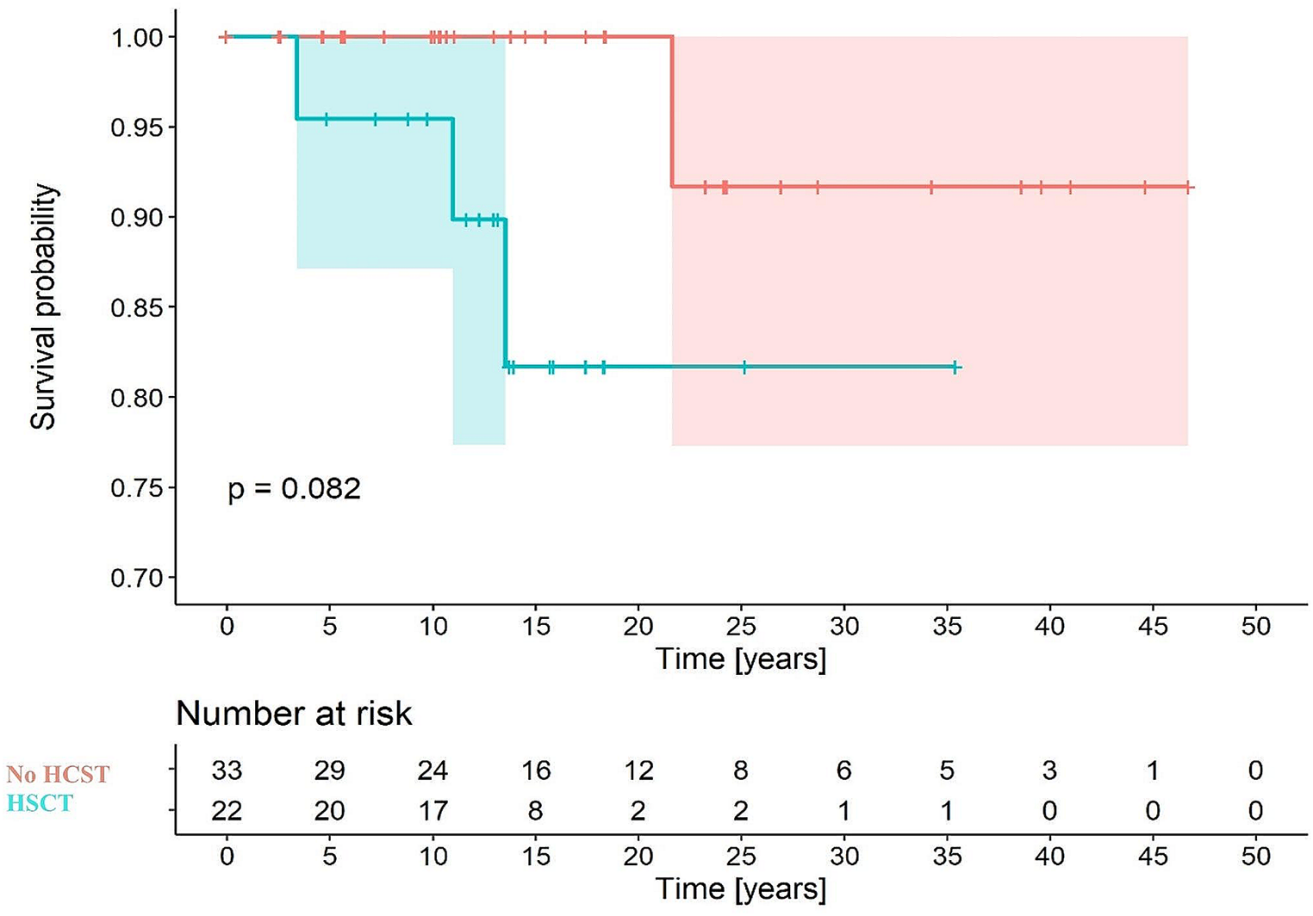
Survival curve in the transfusion-dependent thalassemia (TDT) cohort based on hematopoietic stem cell transplantation (HSCT)

The survival curve based on diagnosis is shown in Fig. 2, with survival at 18 years of 93% for TDT and 100% for NTDT. Figure 3 shows the survival curve depending on whether a hematopoietic stem cell transplant (HSCT) was performed or not. The analysis revealed no significant differences (82% and 92%, respectively).

Viral hepatitis was detected in 9.7% of the cohort. No patients were HIV-infected.

The siblings of 66 of the patients (64.1%) underwent the HLA compatibility study, with 100% compatible healthy donors in 30 cases (45.4%). Of these, 5 (16.6%) did not undergo HSCT, in 2 cases owing to emigration to another country.

Regarding management, 80 patients (77.7%) started chelation treatment during follow-up, with deferasirox being the most widely used agent, followed by deferoxamine and deferiprone (Table 3). When choosing the chelating drug, deferasirox was preferred as the first option in 60.8% and deferoxamine in 33.8%, with a median age of onset of 2.7 years. As second-line treatment, deferasirox was also the most common agent (52.8%), followed by deferiprone (25.0%). Initiation of a second chelating drug was necessary in 42.5% of patients, and a third in 13.7%, either simultaneously or staggered, at a median age of 6.2 and 13.8 years, respectively.

**Table 3:** *HBB* mutations registered in the cohort of patients with non–transfusion-dependent β-thalassemia (NTDT)

Classical nomenclature	HGVS nomenclature	Allelic phenotype	Homozygous state	Heterozygous state	Total mutated alleles	Allele frequency (%)
Codon 39 (C->T)	*HBB*:c.118 > T	β^0^	3	8	14	22.2
IVS-I-6 (T->C) (Portuguese type)	*HBB*:c.92 + 6T > C	β^+^	6	1	13	20.6
Spanish (deltabeta)^0^-Thal	*NG_000011.10:g.5144331_5237241del*	β^0^	3	2	8	12.7
Codons 41/42 (-TTCT)	*HBB*:c.126_129delCTTT	β^0^	0	3	3	4.8
Hb E	*HBB*:c.79G > A	E	0	3	3	4.8
Codons 8/9 (+ G)	*HBB*:c.27_28insG	β^0^	1	0	2	3.2
IVS-I (-3) or codon 29 (C > T)	*HBB*:c.90 C > T	β^+^	1	0	2	3.2
Codon 6 (-A)	*HBB*:c.20delA	β^0^	1	0	2	3.2
CAP + 1 (A > C) silent	*HBB*:c.-50 A > C	β^+^	1	0	2	3.2
IVS-I-110 (G > A)	*HBB*:c.93-21G > A	β^+^	0	2	2	3.2
IVS-I-1 (G > A)	*HBB*:c.92 + 1G > A	β^0^	0	2	2	3.2
-29 (A > G)	*HBB*:c.-79 A > G	β^+^	0	2	2	3.2
Codon 5 (-CT)	*HBB*:c.17_18delCT	β^0^	0	1	1	1.6
Codon 8 (-AA)	*HBB*:c.25_26delAA	β^0^	0	1	1	1.6
IVS-I-5 (G > C)	*HBB*:c.92 + 5G > C	β^+(severe)^	0	1	1	1.6
IVS-II-745 (C > G)	*HBB*:c.316-106 C > G	β^+^	0	1	1	1.6
-88 (C > T)	*HBB*:c.-138 C > T	β^+^	0	1	1	1.6
CAP + 1570 (T > C)	*HBB*:c*96T > C	β^+^	0	1	1	1.6
Poly A (A > G) AATAAA > AATAAG	*HBB*:c.*113A > G	β^+^	0	1	1	1.6
Poly A (A > G) AATAAA > AATGAA	*HBB*:c.*111A > G	β^+^	0	1	1	1.6

HGSV: Human Genome Variation Society.

Thirty-seven patients were treated with vitamin D at some point during follow-up (35.9%); of these, 81.1% maintained therapy. Of the patients who maintained vitamin D, 66.6% were under 18 years of age. This prophylaxis is started at a median of 5.7 years (1.2, 12.8) [0.0-37.8] and is maintained for a median of 4.5 years (1.4, 8.7) [0.0–13.0]. The indication to start treatment with vitamin D was mainly osteopenia, and, less frequently, hypovitaminosis D (resulting from reduced sun exposure) or prophylaxis (65.2% vs. 31.2%, *p* = 0.003).

Therapy with hydroxyurea was initiated in 2.9% of patients during follow-up, before being discontinued in all cases, with a median duration of 8.6 years (8.5, 12.3) [8.3–16.0]. A central venous catheter (CVC) was implanted in 48.5%; this was placed at a median age of 2.9 years (0.8, 12.1) [0.4–35.4]. Splenectomy was indicated in 16 patients (15.5%) at a median age of 9.2 years (6.3, 12.0) [0.8–38.3]. When the sample was divided into 4 cohorts by age in decades and the age at which splenectomy was performed, we observed a statistically significant trend (*p* = 0.054), namely, that in patients diagnosed in the most previous decades, splenectomy was performed earlier and more frequently (Table 4) than in those with a recent diagnosis. Cholecystectomy was performed in 5 patients (4.9%) at a median age of 17.5 years (15.6, 20.2) [11.7–23.8] and with a median interval between diagnosis of cholelithiasis and surgery of 1.0 year (0.7, 1.1) [0.0-1.3]. Of the 3 patients who underwent splenectomy and cholecystectomy, only 1 underwent both procedures in the same operation.

**Table 4:** Chelation therapy in transfusion-dependent thalassemia (TDT) and non–transfusion-dependent thalassemia (NTDT)

Phenotype	TDT (*n* = 103)	NTDT (*n* = 65)
Chelation treatment (%) DFX DFO DFP	80 (77.7) 71 (88.7) 47 (58.7) 18 (22.5)	23 (35.4) 23 (100.0) 6 (26.1) 6 (26.1)
Number of chelators used^1^: 1 2 3	35 (43.7) 34 (42.5) 11 (13.7)	14 (60.9) 6 (26.1) 3 (13.0)
Age at first chelator	2.7 (1.9, 7.0) [1.2–37.1]	24.4 (11.9, 39.2) [2.2–55.7]
First chelator: DFX DFO DFP	60.8% 33.8% 5.4%	77.3% 18.2% 4.5%
Age at second chelator	6.2 (2.8, 12.6) [1.5–38.3]	34.6 (26.3, 49.1) [5.8–56.2]
Second chelator: DFX DFO DFP	52.8% 22.2% 25.0%	50.0% 0.0% 50.0%
Age at third chelator	13.8 (8.9, 20.2) [6.2–33.9]	50.9 (29.9, 55.0) [8.8–59.2]
Treatment duration DFX DFO DFP	6.3 (3.0, 13.4) [0.2–40.2] 6.3 (3.3, 10.6) [0.2–24.9] 7.2 (2.9, 16.0) [0.0-40.2] 1.3 (0.6, 2.8) [0.2–11.3]	3.6 (2.1, 7.6) [0.1–16.4] 3.6 (1.5, 6.4) [0.1–13.4] 5.1 (2.6, 9.7) [2.3–16.4] 0.6 (0.3, 1.9) [0.0-3.3]

Data for qualitative variables are expressed as absolute frequency and/or percentage. Quantitative variables are expressed as the median (interquartile range) and [data range].

DFX: deferasirox.

DFO: deferoxamine.

DFP: deferiprone.

^1^Used simultaneously or staggered.

Forty-one patients underwent HSCT at a median age of 6.4 years (4.4, 9.5) [0.7–16.4]. Five patients (12.2%) presented (or still present) signs and symptoms of chronic graft-versus-host disease. At the time of the data analysis, 51.5% of the transplant recipients presented complete chimerism, while 24.2% presented mixed chimerism without the need for transfusion. Eight patients experienced graft rejection; half of these received a second HSCT.

### Non-transfusion-dependent thalassemia

Most of the patients were diagnosed with clinical anemia (55.4%), while only 7.7% were diagnosed thanks to neonatal screening for SCD (detection of elevated HbA2 and HbF). Diagnosis was through the family study in 15.4%, which was much more frequent in the pediatric cohort than in the adult cohort (*p* < 0.001), where the symptoms of anemia were more frequent (*p* = 0.049). Table 5 shows the mutations in the β-globin (*HBB*) gene detected in the patients who underwent a genetic study (63.1% of the cohort), highlighting a total of 20 mutations. The genetic study was not considered necessary in 24 cases (36.9%). Deletions affecting α-globin genes were found in 17 of the 29 patients in whom the study was carried out (58.6%). No cases of G6PH deficiency were found in the patients evaluated (17.2%). Thrombophilia studies were performed in 20.3% of the cohort, with mutations detected in 4 patients (30.8%).

**Table 5:** Changes in frequency of and age at splenectomy by age cohort in the β-thalassemia cohort (*p* = 0.054)

Age at splenectomy (years)	Age cohorts				
	0–10 years (%)	11–20 years (%)	21–30 years (%)	31–40 years (%)	Total (%)
0–10 years	0 (0.0)	3 (100.0)	7 (77.7)	5 (29.4)	15 (51.7)
11–20 years	-	0 (0.0)	2 (22.3)	2 (11.8)	4 (13.8)
21–30 years	-	-	0 (0.0)	5 (29.4)	5 (17.2)
31–40 years	-	-	-	5 (29.4)	5 (17.2)
TOTAL (%)	0 (0.0)	3 (100.0)	9 (100.0)	17 (100.0)	29 (100.0)

As for loss to follow-up, 60% of patients changed their follow-up center, and 20% emigrated to another country.

Of the 65 patients with NTDT, 4.6% had or had had viral hepatitis, although there were no cases of HIV infection.

The siblings of 15 patients (23.1%) underwent HLA compatibility studies, with identical compatibility in 2 cases; 1 of these 2 patients did not undergo HSCT.

Twenty-three patients started chelation treatment (35.4%). The first choice was deferasirox (73.9%), although all patients used it at some point in the course of their disease (Table 3). Vitamin D3 prophylaxis was started in 26.1% of patients at a median of 31.6 years (12.1, 46.7) and lasted a median of 4.7 years (2.6, 9.4). No differences were observed between the prescription of vitamin D prophylaxis and the size of the centers stratified based on the number of patients in follow-up. As in the TDT patients, differences were observed between patients who take vitamin D depending on whether they had osteopenia or not (76.9% vs. 17.3%, *p* < 0.001).

Thirteen patients (20.0%) took hydroxyurea during follow-up. Of these, 23.1% continue to take the drug. Therapy was initiated at a median age of 12.2 years (5.8, 27.2) and lasted a median of 10.7 years (7.7, 14.5). A CVC was implanted in 4.6% at a median age of 5.7 years (4.0, 10.7). All the CVCs were implanted in 2017. Splenectomy was performed in 15 patients (23.1%) at a median age of 23.0 years (9.4, 30.8), indicating a statistically significant trend with respect to the age of splenectomy in patients with TDT (9.2 years, *p* = 0.052). Seven patients (10.8%) underwent cholecystectomy at a median age of 23.0 years (18.0, 31.7) and with a median interval between diagnosis of cholelithiasis and surgery of 2.9 years (0.9, 3.3). As in the case of the TDT cohort, both splenectomy and cholecystectomy were performed during the same operation in only in 1 of the 3 patients.

One patient (1.5%) with a diagnosis of δβ^0^-thalassemia/β^0^thalassemia underwent HSCT at the age of 8.2 years. Chimerism was complete at the time of data analysis.

### Complications and follow-up

The main complications and the age at presentation are shown in Table 6. A total of 76 patients in the TDT cohort presented at least 1 complication (73.8%), and 72.0% of these patients had more than 3 complications (49 patients presented 3–6 complications and 6 patients more than 6 complications during follow-up). As shown in Table 6, the most frequent complications were hemosiderosis and osteopenia-osteoporosis. In the NTDT group, 46.1% presented at least 1 complication, and 70.0% of these patients presented more than 3.

**Table 6:** Complications in transfusion-dependent thalassemia (TDT) and non-transfusion-dependent thalassemia (NTDT)

	TDT (*n* = 103)		NTDT (*n* = 65)	
Type of comorbidity	n (%)	Age (years)	n (%)	Age (years)
Hemosiderosis	64 (62.1)	3.2 (1.8, 8.0) [0.8–19.4]	18 (27.7)	26.1 (15.7, 37.9) [4.7–50.0]
Osteopenia/osteoporosis	23 (22.3)	16.7 (14.9, 29.4) [6.6–37.0]	13 (20.0)	35.6 (26.6, 48.0) [9.8–55.2]
Hormonal alterations	17 (16.5)	11.4 (6.5, 14.2) [3.5–41.6]	4 (6.1)	12.1 (10.9, 12.8) [8.1–13.9]
Glucose intolerance or diabetes	11 (10.7)	7.7 (5.4, 12.7) [3.5-45.6]	0 (0.0)	
Hypogonadism	9 (8.7)	12.3 (11.5, 15.6) [9.0-41.6]	4 (6.1)	12.3 (12.0, 12.8) [11.8-13.9]
Thyroid hormone alteration		12.3 (12.2, 14.0) [12.1-15.6]	0 (0.0)	
Cortisol alteration	3 (2.9)		1 (1.5)	8.1 (8.1, 8.1) [8.1-8.1]
	0 (0.0)			
Alloimmunization	14 (13.6)	6.4 (4.0, 29.4) [1.8–37.8]	5 (7.7)	7.0 (5.5, 23.3) [3.8–38.6]
Height < 2 DS	13 (12.6)	11.4 (7.9, 12.8) [0.9–16.1]	2 (3.1)	1.8 (0.9, 2.7) [0.0-3.6]
Hypertension/kidney disease	12 (11.6)	14.7 (6.5, 16.9) [4.4–38.8]	2 (3.1)	49.7 (44.0, 55.4) [38.2–61.1]
Viral hepatitis	10 (9.7)	1.4 (0.9, 2.7) [0.9–23.9]	3 (4.6)	3.0 (2.5, 4.0) [2.0–5.0]
Cholelithiasis	9 (8.7)	16.4 (12.7, 22.6) [7.5–38.0]	13 (20.0)	30.4 (18.0, 39.6) [12.0-47.4]
Heart disease	7 (6.8)	21.0 (15.4, 34.5) [10.9–41.5]	6 (9.2)	27.7 (4.0, 43.8) [0.0-50.9]
Thrombosis	3 (2.9)	18.0 (9.3, 25.1) [0.7–32.3]	4 (6.2)	37.3 (30.9, 41.0) [22.0-42.2]
Stroke	1 (1.0)	4.0 (4.0, 4.0) [4.0–4.0]	0 (0.0)	
Pulmonary hypertension	1 (1.0)	28.9 (28.9, 28.9) [28.9–28.9]	2 (3.1)	32.8 (24.5, 41.0) [16.2–49.3]
Hyperhemolytic syndrome	0 (0.0)		2 (3.1)	6.9 (6.2, 7.6) [5.5–8.3]

Data for qualitative variables are expressed as absolute frequency and percentage. Quantitative variables are expressed as the median (interquartile range) and [data range]

SD: standard deviation

In the univariate analysis performed for different variables (Table 7), age (*p* < 0.01) and splenectomy (*p* = 0.002) increased the risk of experiencing at least 1 complication, while diagnosis of NTDT (*p* < 0.001) and deletion of at least 1 α-globin gene (*p* = 0.016) acted as protective factors. In the multivariate analysis (Table 8), statistically significant differences continued to be recorded for diagnosis of NTDT as a protective factor and age or splenectomy as risk factors.

**Table 7:** Univariate analysis: complications by risk factor

Variable	OR	95% CI	p-value
Sex			
Male	-	-	
Female	1.19	0.64, 2.24	0.6
Diagnosis			
TDT	-	-	
NTDT	0.30	0.16, 0.58	< 0.001
Age at diagnosis			
0–1 years	-	-	
1–2 years	2.35	0.85, 7.64	0.12
2–3 years	2.11	0.49, 14.6	0.4
> 3 years	0.43	0.20, 0.92	0.030
Age			
0–5 years	-	-	
6–11 years	8.67	1.93, 62.5	0.011
12–17 years	15.20	3.51, 107.0	0.001
18–24 years	27.3	5.47, 217.0	< 0.001
25–45 years	13.5	3.12, 96.0	0.002
> 46 years	11.4	2.38, 85.8	0.006
Splenectomy			
No	-	-	
Yes	7.06	2.36, 30.5	0.002
Deletion or mutation alpha genes			
No	-	-	
Yes	0.29	0.10, 0.78	0.016

OR: odds ratio.

95% CI: 95% confidence interval.

**Table 8:** Multivariate analysis: complications by risk factor

Variable	OR	95% CI	p-value
Diagnosis			
TDT	-	-	
NTDT	0.15	0.06, 0.36	< 0.001
Age			
0–5 years	-	-	
6–11 years	12.1	2.42, 94.1	0.005
12–17 years	22.7	4.73, 173.0	< 0.001
18–24 years	40.0	6.77, 368.0	< 0.001
25–45 years	15.9	2.99, 129	0.003
> 46 years	20.5	3.19, 190	0.003
Splenectomy			
No	-	-	
Yes	7.81	2.18, 38.2	0.004

OR: odds ratio.

95% CI: 95% confidence interval.

**Fig. 4:**
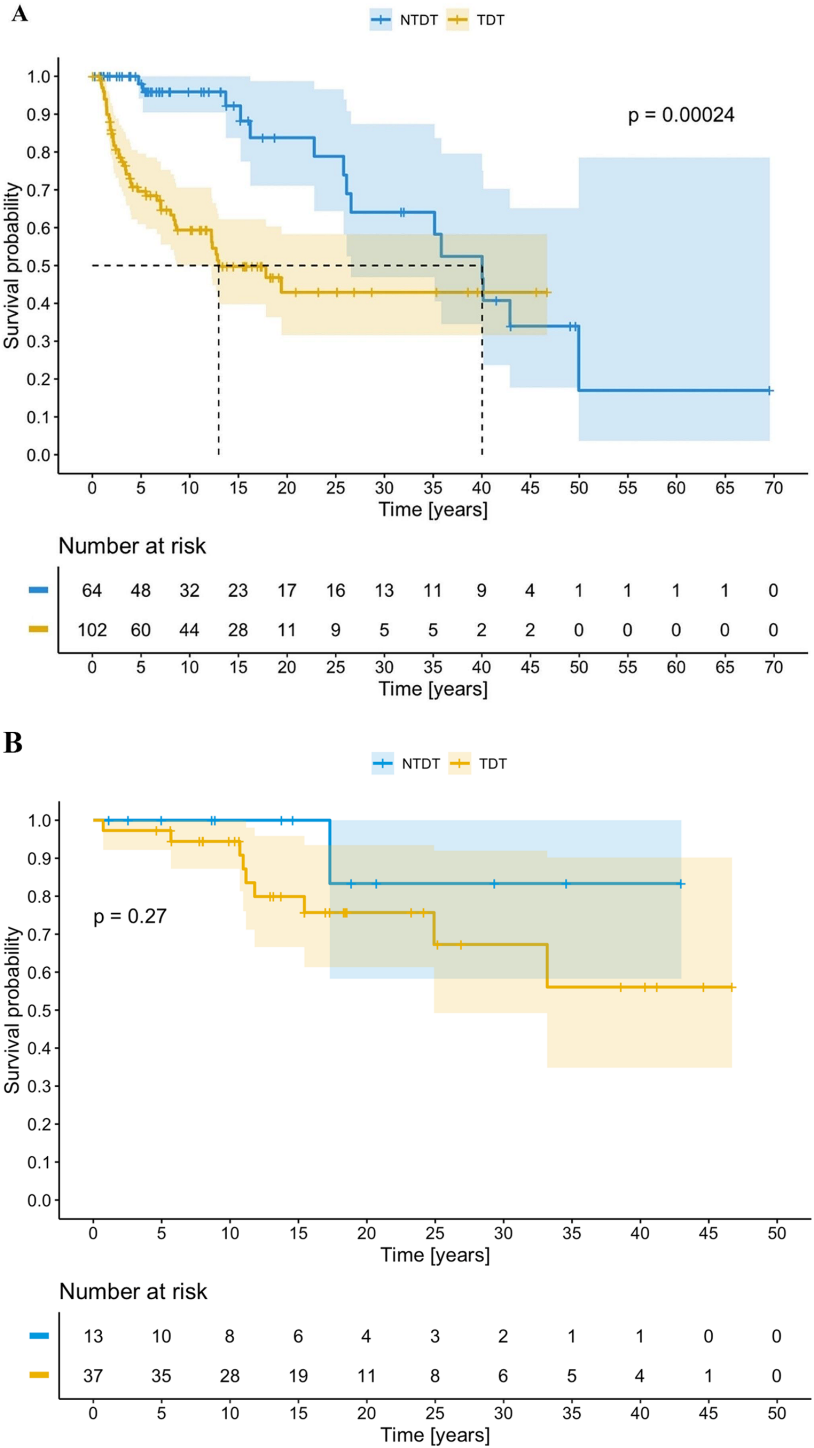
Kaplan-Meier curves for the diagnosis of hemosiderosis (**A**) and a pathological findings on T2* cardiac MRI (**B**) depending on the diagnosis

The Kaplan-Meier curves for the diagnosis of hemosiderosis and pathological outcome on T2* cardiac MRI are shown in Fig. 4.

Regarding the MRI performed in the last 6 years of follow-up of the TDT cohort, 15.5% of patients had only 1 scan, 51.5% never had a scan, and only 1.9% underwent MRI to measure hepatic iron overload annually. In the case of patients with NTDT, only 1 MRI was performed in 24.6% in the last 6 years, while 55.4% never had a scan, and only 9.2% had at least 1 hepatic overload MRI performed every 2 years. A liver iron concentration > 36 µmol/g was recorded in 72% of patients with TDT who had undergone MRI, increasing to 78% in the cohort with NTDT. Overall, up to 25% of patients had evidence of severe hepatic iron overload, with > 190 µmol/g. Regarding T2* cardiac MRIs, only 4.8% of patients in the TDT cohort had an annual scan in the last 6 years of follow-up, with most undergoing only 1 in that time (11.6%). Slightly more than two-thirds (68.9%) never underwent cardiac MRI. Of all the cardiac MRIs performed, only 5.3% of the TDT patients and none of the NTDT patients presented severe myocardial iron overload (< 9 ms), whereas 32.9% and 31.8% had a moderate overload (10–19 ms), respectively.

Of the overall sample, 43 patients (25.6%) were lost to follow-up: 13 patients (30.2%) emigrated to other countries, 21 (48.8%) changed centers, and 5 patients died (3.0% of the entire sample). The probability of survival was 93% at the end of the study period, with an average follow-up of 8.72 years (4.08, 15.52). The causes of death are shown in Table 9.

**Table 9:** Death in the sample: age at death and causes

Patient	Phenotype	Age of death (year)	Cause
1	TDT	3 years (2010)	Sepsis
2	TDT	15 years (1999)	Cardiac hemochromatosis
3	TDT	10 years (1999)	Cardiac hemochromatosis
4	TDT	22 years (2006)	Cardiac hemochromatosis
5	NTDT	54 years (2019)	Uncertain, probably due to cardiac hemochromatosis

## Discussion

This work updates and expands data from the TDT and NTDT cohorts of the first official Spanish registry of patients with rare hemoglobinopathies and anemias (REHem-AR) [11, 12], after the inclusion of almost 650 new patients and the addition of another 17 participating centers. The most frequent mutations, the description of complications, and the follow-up evaluation of iron overload are described for the first time in this cohort of patients. In addition, the information we obtained from the registry provides insight into the life expectancy of affected patients.

Without being the main objective of neonatal screening, 12% of patients with TDT/NTDT were diagnosed within this program. Our population is relatively young (75% were aged less than 20 years) owing to the greater implementation of the REHem-AR among pediatricians, and only 12 centers included adult patients. One of the most common causes of loss to follow-up is changing centers, most likely to an adult unit. The move to another center is a key limitation of this type of registry and an additional reason why this type of registry should include pediatricians and physicians who treat adults.

Within the TDT cohort, the median age at the start of chelation treatment is similar to the recommendations of current guidelines [20] (start of chelation after 2 years of age and when the patient has received between 10 and 20 transfusions). While these data are consistent with those of other series [8], 25% of patients with TDT started chelation treatment after age 7, and in 2 of the 3 young patients who died from cardiac hemochromatosis (10 and 22 years), chelation treatment was started later than recommended (7 and 17 years, respectively).

As in the French registry, where 65% of patients had undergone a mutational study of the β globin gene [9], the bulk of the mutations found (62.8%) were of Mediterranean origin. Although in the Turkish registry the 4 most frequent mutations were the same, the mutation at codon 39 was detected in only 5.7% of patients [10]. Genotyping is not universally performed, probably because it is reserved for cases that raise diagnostic doubts, when genetic counseling is sought, or in antenatal or prenatal diagnosis [21]. In recent years, only the Turkish registry has reported genotyping patients with β-thalassemia [10].

HSCT was prescribed as a curative treatment for the disease in 39.8% of the TDT cohort, somewhat higher than in other registries (13.8%) [9]. The HLA-identical sibling is the donor in 61% of cases. The median age at which patients underwent HSCT is similar to that reported by the European Bone Marrow Transplant Society (EBMT) [22], with no cases in our series receiving the transplant beyond 18 years of age. This is in accordance with the recommendations of the EBMT, whose threshold for achieving optimal results in HSCT is 14 years of age, below which overall survival is 90–96% and event-free survival 83–93% [22]. In our sample, there were no statistically significant differences in the survival of patients with TDT depending on whether they underwent HSCT or not, although 4 of the 5 patients who died in the cohort had received transplants. In this case, the source of the stem cells and HLA matching were not studied.

Regular transfusions in TDT patients and increased intestinal absorption of iron in NTDT patients are the main reasons for elevated iron levels [15, 17–19]. Therefore, the most frequent complication in both cohorts is hemosiderosis, as reported in other European registries [23]. While the complication is addressed in treatment guidelines for this disease, it was undertreated in our series. This finding can be explained by probable information bias and because patients are included in the sample who have not yet initiated this type of drug owing to their young age. The frequency of chelation in individuals with NTDT is consistent with some series [9], although it is far from that reported elsewhere in our environment (such as the Greek registry, with 60.9%) [1]. As in other series [8–10], deferasirox is the most widely used drug during follow-up. Because adherence is better with deferasirox, it is the first-choice chelator among patients, centers, and treating physicians [24]. This circumstance could change with the publication of new data on the administration of deferiprone twice daily [25].

Although potentially subject to the same bias as the information on chelation treatment, the percentage of patients with TDT who were followed up for iron overload with T2* liver or cardiac MRI seems insufficient. However, in the latter case, overload data (> 20 ms) were not reported for most patients with TDT or NTDT. Only 20.4% of the TDT patients and 11.6% of the NTDT patients underwent at least 1 liver or cardiac MRI every 2 years in the last 6 years, potentially indicating poorer-quality management. Of note, these percentages are based on the historical cohort, without taking into account the patients who were lost to follow-up, indicating that the real figures are close to the data—also insufficient—reported by other registries [8, 9]. Only the combination of serum ferritin levels with hepatic and cardiac T2* MRI studies has proven to have a strong prognostic value and an adequate predictive value for the development of complications, although observational studies continue to confirm a correlation between ferritin levels and liver iron concentration in NTDT patients [19].

Almost half of the TDT patients had a CVC. This is especially useful in younger children since it allows rapid intravenous access in cases of acute complications and prevents repeated venipuncture, especially in patients who do not have easy vascular access. In addition, several studies support the safety of these devices, with a low incidence of infectious, thrombotic, and mechanical complications [26].

Our splenectomy data are far from the percentages reported in other series [1, 9, 10, 23]. One possible reason is that our series is relatively young, so most patients have had “modern” follow-up [17], where a regular and adequate transfusion regimen improves tissue oxygenation [27] and reduces the need for splenectomy, as dictated by current guidelines [17, 19]. Proof of this is that the highest percentages of splenectomy in our cohort are found in individuals ≥ 21 years of age.

Iron overload and viral hepatitis are the two main causes of liver disease in patients with TDT. Infections have even become the leading cause of death in western countries [17]. However, very few patients in our cohort had viral hepatitis compared to other published series [9], possibly owing to the “youth” of our cohort. The fact that the mean age of our patients is much lower than in other series means that most transfusions were performed after the introduction of virus screening in blood donations. Therefore, other series did not find HCV positivity in patients transfused after 1990 [23]. Musculoskeletal diseases (osteopenia, osteoporosis [28, 29]) are the second most frequent group of complications. In the Italian registry [23], these diseases are the most common complication (almost 60% of patients), possibly also due to the older age of the cohort (30.6 ± 7.7 years). Even in a country like Spain, with sunshine for most of the year, the need for primary prophylaxis of hypovitaminosis D is clear [11, 12, 17, 30], thus supporting exhaustive monitoring of these complications owing to the relationship between them from adolescence. Therefore, endocrinological diseases are also relevant complications, more so in patients with TDT, consistent with results reported elsewhere [17–19, 31]. In our sample, hypogonadism was not as frequent, whereas in others it can affect almost 50% of patients with TDT [9] and, more specifically, up to 68% of men and up to 31% of women (in the form of amenorrhea) [17, 19]. In the TDT group, height < 2 SD was similar to the French registry [9]. These findings indicate a reduction in the frequency of this complication, since an adequate and regular transfusion regimen is prioritized in affected patients [32]. To our knowledge, ours is the first registry to report the percentage of alloimmunization in patients with TDT or NTDT. This not insignificant complication, as it can make it difficult to find compatible blood for affected patients. It is also associated with hemolytic reactions, thus making it essential that patients be followed up in referral centers, and highlights the relevance of performing extended erythrocyte phenotyping or genotyping before transfusions [33].

As for identification of risk factors for complications, our study confirms that complications appear at an older age, as reported in the literature [9, 25, 34], although we were unable to demonstrate a linear progression. In addition, while splenectomy is also a risk factor for complications, the practice is increasingly abandoned.

As shown in Table 9, the main cause of death in patients with thalassemia is heart problems however, they have significantly declined [17, 35]. However, the presence of cardiovascular complications in our cohort may seem less common than expected, possibly as a result of advances in recent decades in chelation and monitoring of iron overload [16, 36]. The fact that heart problems are more common in patients with NTDT should make us review management with iron chelators and follow-up with T2* MRI (first MRI at 10 years and initiation of chelation if > 90 µmol/g or serum ferritin level ≥ 800 ng/mL) [19], thus highlighting the need for a more exhaustive approach in this population. As supported by our survival rate, the life expectancy of affected patients has improved over the last 2 decades [8, 17].

Our study is subject to a series of limitations. The rate of loss to follow-up is noteworthy, and there was a certain lack of response in some centers consulted; therefore, the results may have been affected by the data lost for some variables with respect to the total number of patients. This shortcoming should be improved in future updates. The recent addition of adult patients to the registry may have influenced the low number of older patients, although this has been amended since the previous update.

## Conclusions

Our registry enables us to describe the management of β-thalassemia in Spain, as well as to analyze the morbidity and mortality in patients with TDT and NTDT.

Gene therapy continues to be in its early stages. Meanwhile, HSCT continues to be the curative treatment for many severe anemias such as TDT. In recent years, the introduction of T2* sequences in MRIs for the evaluation of myocardial and hepatic iron concentrations has enabled more exhaustive and less aggressive diagnosis of overload and can provide us with information about adherence to chelation treatment. Treatments can therefore be adapted or changed, and educational strategies individualized [8, 37]. This is also why patients must receive multidisciplinary care in specialized centers, where all the necessary tools for their management are available and have access to clinical trials.

Complications related to iron overload in TDT and NTDT account for most of the morbidity and mortality associated with β-thalassemia, and their social, psychological, and economic impacts are considerable. Given the increasing frequency of osteopathy and endocrinological complications, more attention is required to ensure proper management and follow-up. Even so, cardiac complications are the main cause of death. Therefore, regular follow-up with T2* cardiac MRI, together with adequate and adherent chelation treatment, is considered essential to improve survival.

The convenience and simplicity of online records, along with the possibility of homogenizing variables and periodic updating of data, ensures valuable information on these diseases in Spain. In addition, collaboration with European networks recommends their extension to other rare anemias. This approach will favor the development of other studies to obtain important information that can guide the health authorities in improving the needs of affected patients, in terms of both management and follow-up.

## Data Availability

The data that support the findings of this study are available on request from the corresponding author. The data are not publicly available due to ethical restrictions.

## Declarations

S.P.P. has received honoraria for speaking engagements and other activities from Novartis, Agios Pharmaceuticals, and Vertex Pharmaceuticals.

R.B. has received fees for participating in talks and for his participation as a consultant for Novartis. A.R. and R.B. have led a research project partially funded by Novartis.

The study was partially supported by Unidad de Investigación Materno Infantil Fundación Familia Alonso (UDIMIFFA).

## Abbreviations

AEMPS: Agencia Español de Medicamentos y Productos Sanitarios
AEPD: Agencia Española de Protección de Datos
CVA: cerebrovascular accident
CVC: central venous catheter
EBMT: European Society for Blood and Marrow Transplantation
SCD: sickle cell disease
G6PD: glucose 6-phosphodehydrogenase deficiency
Hb: Hemoglobin
HCV: hepatitis C virus
HSCT: hematopoietic stem cell transplantation
MRI: magnetic resonance imaging
NTDT: non–transfusion-dependent thalassemia
REHem-AR: Registro Español de Hemoglobinopatías y Anemias Raras
SCD: sickle cell disease
SD: standard deviation
SEHH: Sociedad Española de Hematología y Hemoterapia
SEHOP: Sociedad Española de Hematología y Oncología Pediátricas
TI: thalassemia intermedia
TDT: transfusion-dependent thalassemia
TM: thalassemia major
