# Correction to: Spanish registry of hemoglobinopathies and rare anemias (REHem- AR): demographics, complications, and management of patients with β-thalassemia

**DOI:** 10.1007/s00277-024-05838-1

**Published:** 2024-06-21

**Authors:** Eduardo J. Bardón-Cancho, José Manuel Marco-Sánchez, David Benéitez-Pastor, Salvador Payán-Pernía, Anna Ruiz Llobet, Rubén Berrueco, Marina García-Morin, Cristina Beléndez, Leonor Senent, María José Ortega Acosta, Irene Peláez Pleguezuelos, Pablo Velasco, Anna Collado, Marta Moreno-Carbonell, Bienvenida Argilés, Inmaculada Pérez de Soto, María del Mar Bermúdez, Eduardo J. Salido Fiérrez, Adoración Blanco-Álvarez, Pablo González Navarro, Elena Cela

**Affiliations:** 1 Data Manager de Grupo de trabajo de Eritropatología de la Sociedad Española de Hematología y Oncología Pediátricas (SEHOP), Hospital General Universitario Gregorio Marañón, Calle O’Donnell, 48, Madrid, España; 2 Sección de Hematología y Oncología Pediátricas, Servicio de Pediatría, Hospital General Universitario Gregorio Marañón, Instituto de Investigación Sanitaria Gregorio Marañón, CSUR Eritropatología, ERN-EuroBloodNet. CIBERER, Facultad de Medicina, Universidad Complutense de Madrid, Madrid, España; 3 Grupo de Investigación Translacional en Anemias Minoritarias, Unidad de Eritropatología, Servicio de Hematología Clínica, Hospital Universitario Vall d’Hebron, Vall d’Hebron Institut de Recerca (VHIR) y Vall d’Hebron Institut d’Oncologia (VHIO), ERN-EuroBloodNet. CIBERER, Universitat Autònoma de Barcelona, Grupo de Eritropatología SEHH, Grupo Clínico Vinculado GCV21/ ER/1, Barcelona, España; 4 Servicio de Hematología. Hospital Universitario Virgen del Rocío, Instituto de Biomedicina de Sevilla (IBIS)-Consejo Superior de Investigaciones Científicas (CSIC), Universidad de Sevilla, Sevilla, España; 5 Servicio de Hematología Pediátrica. Hospital Sant Joan de Déu Barcelona, Institut de Recerca Pediàtrica Hospital San Joan de Déu de Barcelona (IRP-HSJD), Universitat de Barcelona, Esplugues de Llobregat, Barcelona, España; 6 Servicio de Hematología, Hospital Universitario y Politécnico La Fe, Valencia, España; 7 Servicio de Hematología Infantil, Hospital Universitario Virgen de las Nieves, Granada, España; 8 Servicio de Hematología Infantil, Hospital Universitario Vall d’Hebron, Barcelona, España; 9 Servicio de Hematología. Hospital General Universitario Gregorio Marañón, Instituto de Investigación Sanitaria Gregorio Marañón, CSUR Eritropatología. ERN-EuroBloodNet, Universidad Complutense de Madrid, Madrid, España; 10 Servicio de Hematología Infantil, Hospital Universitario y Politécnico La Fe, Valencia, España; 11 Servicio de Oncología Pediátrica, Hospital Virgen de la Arrixaca, Murcia, España; 12 Servicio de Hematología, Hospital Virgen de la Arrixaca, Murcia, España; 13 Unitat de Genètica Molecular Hematològica. Servei d’Hematologia, Hospital Universitari Vall d’Hebron, Murcia, España; 14 Bioestadístico, Unidad de Investigación Materno InfantilFundación Familia Alonso (UDIMIFFA), Instituto de Investigación Sanitaria Gregorio Marañón (IiSGM), Hospital General Universitario Gregorio, Madrid, España

**Correction to: Annals of Hematology**

10.1007/s00277-024-05694-z

The authors want to clarify that the correct name of the co-author Marta Moreno instead of Marta Moreno-Servet is Marta Moreno-Carbonell.

The original article has been corrected.

